# Targeting USP14 sensitizes glioma to radiotherapy through exacerbated reactive oxygen species accumulation

**DOI:** 10.4103/mgr.MEDGASRES-D-26-00027

**Published:** 2026-09-12

**Authors:** Xinyu Liu, Lichen Shen, Yijun Lu, Zimeng Wang, Shuanghu Yuan, Hongcang Gu, Junchao Qian

**Affiliations:** 1 Institute of Physical Science and Information Technology, Anhui University, Hefei, Anhui Province, China; 2 Anhui Province Key Laboratory of Medical Physics and Technology, Institute of Health and Medical Technology, Hefei Institutes of Physical Science, Chinese Academy of Sciences, Hefei, Anhui Province, China; 3 Department of Radiation Oncology, The First Affiliated Hospital of USTC, Division of Life Sciences and Medicine, University of Science and Technology of China, Hefei, Anhui Province, China; 4 Department of Radiation Oncology, Anhui Provincial Cancer Hospital, Hefei, Anhui Province, China

**Keywords:** apoptosis, deubiquitinating enzyme, DNA damage, endoplasmic reticulum stress, glioma radioresistance, glioma, radiosensitization, radiotherapy, reactive oxygen species, USP14

## Abstract

Radiotherapy is one of the most effective treatments for glioma, but therapeutic efficacy is strongly limited due to intrinsic radioresistance, which is closely linked to reactive oxygen species. This research was designed to elucidate the role and underlying mechanisms of the deubiquitinase ubiquitin-specific protease 14 (USP14) in reactive oxygen species Accumulation and radioresistance of glioma. Bioinformatics analysis demonstrated that upregulation of USP14 was associated with tumor progression, high levels of USP14 in glioma patients were correlated with a significantly poorer prognosis. Cytological experimental results demonstrated that radiotherapy could induce increased USP14 expression. Inhibition of USP14 disrupted intracellular protein homeostasis, leading to upregulation of endoplasmic reticulum stress-related proteins thereby exacerbating endoplasmic reticulum stress. This stress response further induced substantial reactive oxygen species generation and DNA damage accumulation, ultimately synergistically enhancing the tumoricidal effect of radiotherapy. *In vivo*, the combined application of b-AP15 (a USP14 inhibitor) and radiotherapy elicited potent antitumor effects, marked by significant suppression of tumor growth and increased apoptotic cell death. In summary, we reveal a new regulatory axis by which USP14 governs glioma radiosensitivity through reactive oxygen species-mediated DNA damage and apoptosis, providing a promising therapeutic target and strategy for reversing clinical radioresistance.

## Graphical abstract

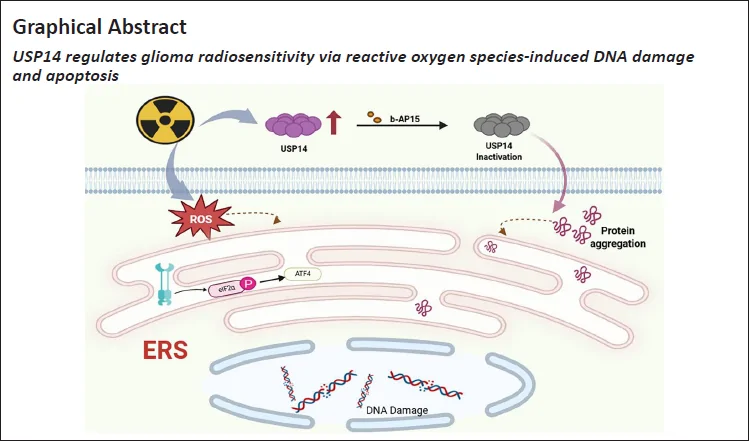

## Introduction

The most common and invasive primary brain malignancy tumor in adults is glioma.^1^ The therapies used as first-line treatment, such as surgery, radiation and systemic chemotherapy, cannot prolong the median life of patients up to 15 to 20 months.^2^,^3^ Radiotherapy, which is one of its pillars and most effective modalities in clinical practice, destroys the remaining tumor cells and increases progressions free interval. Nevertheless, increased radioresistance often contributes to the failure of treatment, recurrence at local sites, and deplorable patient prognoses, which is a significant setback to enhancing the effectiveness of treatment.^4^ Consequently, the demonstration of molecular mechanisms underlying radioresistance of glioma and the elaboration of applicable radiosensitizing treatment approaches are important scientific concerns to enhance the outcomes in the prognosis of patients.

Ionizing radiation exerts its cytotoxic effects primarily through the induction of DNA damage, most notably via the generation of double-strand breaks.^5^ A principal mechanism underlying this damage involves the production of reactive oxygen species (ROS), which directly target DNA bases and the sugar-phosphate backbone, leading to the formation of complex clustered lesions with limited reparability.^6^ Nevertheless, the use of ROS in radiotherapy is 2-fold. Although radiotherapy-induced ROS play an important role in tumor killing, glioma cells also adopt an adaptive reaction to alleviate oxidative stress.^7^ These defense mechanisms encompass the upregulation of endogenous antioxidant systems and the enhanced clearance of oxidatively damaged proteins through proteolytic degradation pathways.^8^,^9^ These adaptations decrease ROS-mediated lethality, enhance survival, and also determine a state of radioresistance. Consequently, therapeutic interventions aimed at interfering with this adaptive redox homeostasis by inhibiting important antioxidant enzymes or disrupting the clearance of damaged proteins to increase the level of ROS during radiotherapy, can be viewed as one of the rational and mechanistically based approaches to radiation resistance.

The ubiquitin-proteasome system is responsible for maintaining cellular protein homeostasis by selectively degrading misfolded, damaged, or obsolete proteins.^10^ Its role can also be applied to the regulation of oxidative stress because it regulates the turnover of the central elements in the antioxidant and pro-oxidant circuits.^11^ Ubiquitin specific peptidase 14 (USP14), an essential deubiquitinating protease, removes ubiquitin chains on its targets to regulate protein stability and affect a wide range of processes, including proliferation and stress response.^10^,^12^ In glioma, USP14 has been involved in tumorigenesis and resistance to drugs, and recently, it has been correlated with radiosensitivity through DNA repair regulation.^13^ Although existing studies have implicated USP14 in treatment resistance, whether it specifically regulates radiosensitivity through the modulation of ROS metabolism remains to be elucidated.

Therefore, we propose the role of the USP14 in glioma and its effects on radiosensitivity. We begin by determining the clinical prognostic value of USP14 and its association with radiotherapy response. Afterwards, consecutive experiments were conducted to determine the effect of inhibition of USP14 on the radio sensitivity of glioma cells. The potential mechanisms were illuminated through a comprehensive investigation of various aspects, including the modulation of endoplasmic reticulum stress (ERS), the generation of ROS, and the accumulation of DNA damage.

## Methods

### Bioinformatics analysis

USP14 expression profiles in normal brain, low-grade glioma, and glioblastoma tissues were analyzed using the Gene Expression Profiling Interactive Analysis (GEPIA) database (http://gepia.cancer-pku.cn)^14^ with default parameters. Survival curves were generated using the Kaplan-Meier plotter (http://kmplot.com)^15^ and the Chinese Glioma Genome Atlas (http://www.cgga.org.cn).^16^ Patients were divided into high- and low-USP14 expression groups based on the median expression level. For radiotherapy-subgroup analysis, only patients who had received radiotherapy were included. The log-rank test was used to compare survival differences, and hazard ratios with 95% confidence intervals were calculated by univariate Cox regression. A *P*-value < 0.05 was considered statistically significant.

### Cell culture

The rat glioma C6-Luc cell line (Lot No. LZQ0003) was obtained from Shanghai Zhong Qiao Xin Zhou Biotechnology Co., Ltd. (Shanghai, China) and authenticated by short tandem repeat profiling. Cells were cultured at 37°C, 5% CO_2_. The culture medium consisted of Ham’s F-12K (Procell, Wuhan, China, PM150912) supplemented with 15% horse serum (Procell, 164215), 2.5% fetal bovine serum (CLARK Bioscience, Webster, TX, USA, FB25015), and 1% penicillin/streptomycin (Hyclone, Logan, UT, USA, SV30010).

### Irradiation and drug treatment

The cells were exposed to 8 Gy radiation by a 6 MV medical linear accelerator (Elekta Axesse, Stockholm, Sweden). Subsequently, the cells were incubated at 37°C in a 5% CO_2_ incubator with penicillin–streptomycin-supplemented media.

For drug treatment, cells were incubated with the USP14 inhibitor b-AP15 (MedChemExpress, Monmouth Junction, NJ, USA, HY-13989) at indicated concentrations (10 or 20 μM) for 24 hours. In combination experiments, cells were treated with b-AP15 immediately after irradiation and then co-incubated for 24 hours. Control cells received an equivalent volume of vehicle (dimethyl sulfoxide, final concentration < 0.1%).

### Establishment of glioma model

The specific pathogen-free male Sprague-Dawley rats (8 weeks old, 220–250 g) were provided by Vital River. All animal procedures were conducted with the approval of the Institutional Animal Care and Use Committee of Hefei Institutes of Physical Science, Chinese Academy of Sciences (approval No. DWLL(E)-2025-42, approved on May 8, 2025). All experimental procedures strictly adhered to the Guide for the Care and Use of Laboratory Animals, 8^th^ ed.^17^

Rats were randomly assigned to four treatment groups (*n* = 3 per group): PBS control, b-AP15 monotherapy (3 mg/kg, intraperitoneal injection), radiotherapy alone, and b-AP15 combined with radiotherapy. For the radiotherapy groups, a dose of 8 Gy was delivered to the brain region using a 6 MV medical linear accelerator (Elekta Axesse). Thus, both b-AP15 injection and 8 Gy irradiation were performed three times in total.

After isoflurane (RWD, Shenzhen, China, R510-22-10) anesthesia (4% to induce, 1.5–2.0% to maintain), the rats were injected with log-phase C6 cells (5 × 10^6^ cells/μL) at the striatum (1 mm anterior, 3 mm lateral to Bregma) with a controlled infusion rate of 2 min/μL. The needle was retained for 5 minutes post-injection to minimize reflux. Subsequently, the burr hole was sealed with bone wax and the incision sutured. Finally, the surgical wound was disinfected daily with povidone-iodine for postoperative care. At the end of the experiment, all rats were euthanized by cervical dislocation under isoflurane anesthesia (4%), and tumor tissues were harvested from the brain.

### Measurements of reactive oxygen species levels

The log-phase growth of 8 × 10^4^ cells/well were rinsed by pouring PBS after which they were dark stained for 30 minutes using DCFH-DA at room temperature. The ROS formation was monitored with the help of a fluorescence microscope after the final PBS wash.

To study the tumors on a histological basis, the tumor specifics were embedded into optimal cutting temperature compound and cut at 5 μm. The next sections were then incubated at the temperature of 37°C, with ROS detection solution and once incubated they were then washed, mounted and tested under a fluorescence microscope to obtain an image.

### Cell counting kit-8 assay

C6 cells were loaded in 96-well (1 × 10^3^ cells/well) plates and allowed to grow for 24 hours. Another treatment, cells were incubated with 10 µL of cell counting kit-8 solution (Dojindo, Mashiki, Tabaru, Japan, CK04) at 37°C for 1 hour. With a microplate reader, the optical density was measured at 450 nm. The proliferation (%) = optical density_experimental_/optical density_control_ × 100.

### Western blot analysis

Based on the experimental groups, C6 cells were treated to specific drug treatments and irradiation. After treatment C6 cells were scraped after being washed twice with ice-cold phosphate-buffered saline (PBS). RIPA lysis buffer (Beyotime, Shanghai, China, P0013B) containing a protease and phosphatase inhibitor cocktail on ice were added to lysate cell pellets 30 min and centrifuge at extremely high speed, 12,000 × *g* at 4°C to isolate soluble cell contents. The protein concentration was measured by using bicinchoninic acid protein assay kit (Beyotime, P0012). Proteins were added in an equal amount to 5× sodium dodecyl sulphate loading buffer and boiled at 95°C 10 minutes. Equal quantity of proteins was then separated on 10 percent SDS-PAGE gels and transferred onto PVDF membranes. Following transfer, 3% bovine serum albumin in Tris-buffered saline with Tween 20 at 37°C was used to block membranes after transfer. Primary antibodies against USP14 (rabbit, 1:1000, Wuhan Sanying, Wuhan, China, Cat# 145172-1-AP, RRID: AB_2257124), ubiquitin (rabbit, 1:1000, Wuhan Sanying, Cat# 10201-2-AP, RRID: AB_671515), protein kinase R-like endoplasmic reticulum kinase (PERK; mouse, 1:1000, Wuhan Sanying, Cat# 68482-1-Ig, RRID: AB_3085193), phospho-PERK (p-PERK; rabbit, 1:500, rabbit, MedChemExpress, Cat# HY-P81190, RRID: AB_2257124), eukaryotic initiation factor 2α (eIF2; rabbit, 1:1000, Wuhan Sanying, Cat# 11170-1-AP, RRID: AB_2096489), p-eIF2α (rabbit, 1:1000, Wuhan Sanying, Cat# 28740-1-AP, RRID: AB_2881204), activating transcription factor 4 (ATF4; mouse, 1:1000, Wuhan Sanying, Cat# 60035-1-Ig, RRID: AB_2058598), C/EBP homologous protein (CHOP; rabbit, 1:1000, Wuhan Sanying, Cat# 15204-1-AP, RRID: AB_2292610), and glyceraldehyde-3-phosphate dehydrogenase (GAPDH; mouse, 1:2000, Wuhan Sanying, 60004-1-Ig, RRID: AB_2107436). After Tris-buffered saline with Tween 20 was added, horseradish peroxidase-conjugated secondary antibodies (rabbit, 1:10,000, Zenbio, Durham, NC, USA, Cat# 511103, 511203) were added to the membranes and allowed to incubate at 37°C for 1 hour. Protein bands were visualized using an enhanced chemiluminescence kit (Servicebio, Wuhan, China, G2074) and detected with a chemiluminescence imaging system (Thermo Fisher Scientific, 5200Multi, Waltham, MA, USA). Band intensities were measured by densitometry using ImageJ software (1.51j8; National Institutes of Health, Bethesda, MD, USA).^18^

### Reverse transcription-quantitative polymerase chain reaction analysis

To quantify gene expression via reverse transcription-quantitative polymerase chain reaction, we first isolated total RNA with a commercial kit (TransGen Biotech Co., Ltd., Beijing, China, ER501-01), followed by reverse transcription into complementary DNA using reverse transcription kit (Vazyme R233-01). Polymerase chain reaction amplification proceeded on a real-time cycler (Roche LightCycler96, Basel, Switzerland) according to the following protocol: 5 minutes at 95°C, then 40 cycles of 10 seconds at 95°C and 30 seconds at 60°C. The 2^–ΔΔCT^ method was employed to determine relative expression levels, using GAPDH as the endogenous control. Primer sequences (General Biol, Chuzhou, China) are provided in **Table 1**.

**Table 1:** PCR primer sequences

Gene	Species	Primer sequence (5ʹ – 3ʹ)
*USP14*	Rat	Forward: GGT CAG CAG ATG CTC TTC CA
		Reverse: AAT CCA CAC GGC AAC TCC AT
*PERK*	Rat	Forward: TTT CGG TGC TCC AAG GCT C
		Reverse: GAT GTG GGA GCA AGC AAG C
*EIF2S1*	Rat	Forward: ACA ACG ACC CTA GAG AGG AC
		Reverse: ATC TGT AAC CAC TTT GGG CTC C
*ATF4*	Rat	Forward: GTC TCT CTG TAC GCT GTT CCT
		Reverse: CTG CTG CCT CTA ATA CGC CAT
*CHOP*	Rat	Forward: CGA TCA TAC CAT GTT GAA GAT GAG C
		Reverse: ATG CAC TTC CTT CTG GAA CAC TCT C
*GAPDH*	Rat	Forward: ATT TCA GAT TGG ACC TTG TG
		Reverse: GTG CCG CTT GCT TAG TT

ATF4: Activating transcription factor 4; CHOP: C/EBP homologous protein; EIF2S1: eukaryotic translation initiation factor 2 subunit 1; GAPDH: glyceraldehyde-3-phosphate dehydrogenase; PCR: polymerase chain reaction; PERK: protein kinase R-like endoplasmic reticulum kinase; USP14: ubiquitin-specific protease 14.

### Live/dead staining

For live/dead staining, C6 cells were seeded in 48-well plates and cultured overnight at 37°C for adhesion. This was followed by treatment with b-AP15 (10 μM), immediately after which radiotherapy was administered, and the cells were co-incubated for 24 hours. To determine viability, the cell was incubated in Calcein-AM (green) and propidium iodide (red) (Servicebio, G1707) after 15 minutes. These images were taken under the corresponding excitation wavelengths of 490 nm and 545 nm using a Leica DFC310 FX microscope.

### Detection of reactive oxygen species by flow cytometry

When the treatment was completed, the cells would be collected and incubated with 10 µM DCFH-DA (aladdin, 4091-99-0, Shanghai, China) into 40 minutes at 37°C and then washed three times with PBS. Flow cytometric analysis (CytoFLEX LX, Beckman, Brea, CA, USA, Ex/Em = 488/525 nm) was applied on the samples to measure the ROS. To analyze the data, the relative level of ROS was normalized by the mean fluorescence intensity of control group, as performed under FlowJo software (v7.6. 1, FlowJo LLC, Ashland, OR, USA).

### Analysis of apoptosis by flow cytometry

The apoptosis of C6 cells was analyzed using Annexin V-FITC/propidium iodide dual-staining kit (eBioscience, San Diego, CA, USA). After staining, the samples were analyzed by flow cytometry (Miltenyi Biotec, Auburn, CA, USA), and analyzed using the FlowJo software.

### Immunofluorescence staining

To perform immunofluorescence staining, the tissues (glioma cells and glioblastoma tissue sections in a freezer) were fixed in 4% paraformaldehyde for 15 minutes, permeabilized in 0.1% Triton X-100 for 10 minutes, and blocked in 5% goat serum for 1 hour. At 4°C, the samples were incubated overnight with γ-H2AX antibodies (mouse, 1:100, Zenbio, Cat# 201082-7G9,RRID:AB_2722720) and then washed with PBS and incubated with Alexa Fluor 488 conjugated secondary antibodies (goat anti-mouse, 1:1000, Servicebio, Cat# GB25301, RRID:AB_2904018) for 2 hours at room temperature afterward and with 4’,6-diamidino-2-phenylindole counterstained for (Servicebio, G1012) at 15 minutes. Finally, specimens were fixed on Vectashield antifade mounting medium (Beyotime, H-1200-10) and examined with a fluorescence microscope (Nikon Eclipse Ti-U, Tokyo, Japan).

### Clonogenic assay

The clonogenic survival of cells was evaluated. Cells were seeded and treated in 6-well plates at densities of 500–1000 cells per well. Following fixation and staining with 0.5% crystal violet (in methanol: acetic acid, 75:25) after 14 days of culture, colonies were microscopically quantified. The colonies composed of 50 or more cells were defined as surviving colonies.

### Terminal deoxynucleotidyl transferase dUTP nick-end labeling staining

For the detection of apoptosis, terminal deoxynucleotidyl transferase dUTP nick-end labeling staining was performed on paraffin brain sections. Briefly, sections were dewaxed and rehydrated, then treated with proteinase K (20 µg/mL) at 37°C for 15 minutes and permeabilized with 0.1% Triton X-100. The terminal deoxynucleotidyl transferase dUTP nick-end labeling reaction was carried out by incubating sections with the reaction mixture at 37°C for 1 hour in the dark. Following PBS washes, sections were mounted with 4’,6-diamidino-2-phenylindole-containing mounting medium (Beyotime, H-1200-10) and visualized under a fluorescence microscope.

### Hematoxylin and eosin staining

Glioma tissue sections (4 μm) were baked at 60°C for 2 hours, followed by deparaffinization in xylene (Sinpopharm, Shanghai, China, 10023418) and rehydration (Sinpopharm, 10009218) through a graded ethanol series. Sections were then stained with Harris hematoxylin (Servicebio, G1004) for 5–8 minutes, washed, differentiated, and blued with ammonia (Acros, Geel, Belgium, C390030010). Counterstaining was performed with 0.5% eosin (Servicebio, G1002) for 1–2 minutes. After rapid ethanol dehydration, clearing in xylene, and mounting with neutral gum, the sections were examined under a light microscope (Olympus Bx53, Tokyo, Japan).

### Statistical analysis

Data were statistically analyzed and visualized with GraphPad Prism (version 9.3, GraphPad Software, Boston, MA, USA, www.graphpad.com). Comparisons between two groups were conducted using unpaired Student’s *t*-test. For multiple group comparisons, one- or two-way analysis of variance was employed, followed by Tukey’s multiple comparisons test for *post-hoc* pairwise analysis. All values are reported as mean ± standard deviation (SD). A *P*-value < 0.05 was considered statistically significant.

## Results

### High expression of USP14 is associated with poor prognosis in gliomas

To systematically evaluate the therapeutic potential of USP14 in glioma, we first analyzed to assess the candidacy of USP14 as a therapeutic target on public databases. Analysis using the GEPIA database showed that, compared to normal brain tissue, USP14 shows an upregulation trend in both low-grade and high-grade gliomas (**Figure 2A**). Further survival analysis indicated that elevated USP14 expression correlates significantly with a poor prognosis in glioma, a result also validated in the Chinese Glioma Genome Atlas database (**Figure 2B–D**). Notably, in the subgroup of glioma patients who had received radiotherapy, high USP14 expression similarly predicted shorter survival, suggesting that USP14 may be involved in mediating radioresistance (**Figure 2E** and **F**). As well, Western blot and polymerase chain reaction were used the proofs that the combination of radiotherapy leads to the upregulation of the USP14 expression, which further confirmed its role in cellular radio-adaptive responses (**Figure 2G** and **H**). Overall, these findings suggest that the expression of USP14 is associated with an adverse prognosis among glioma patients, particularly those undergoing radiotherapy, which suggests that USP14 could mediate the patient outcomes due to its potential to drive the emergence of radioresistance.

**Figure 1:**
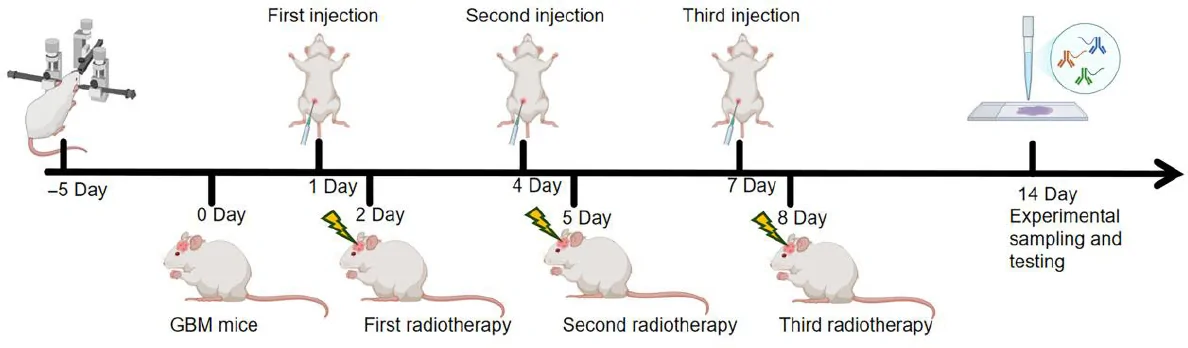
Flowchart of *in vivo* experiments. GBM: Glioblastoma. Created with BioRender.com.

**Figure 2:**
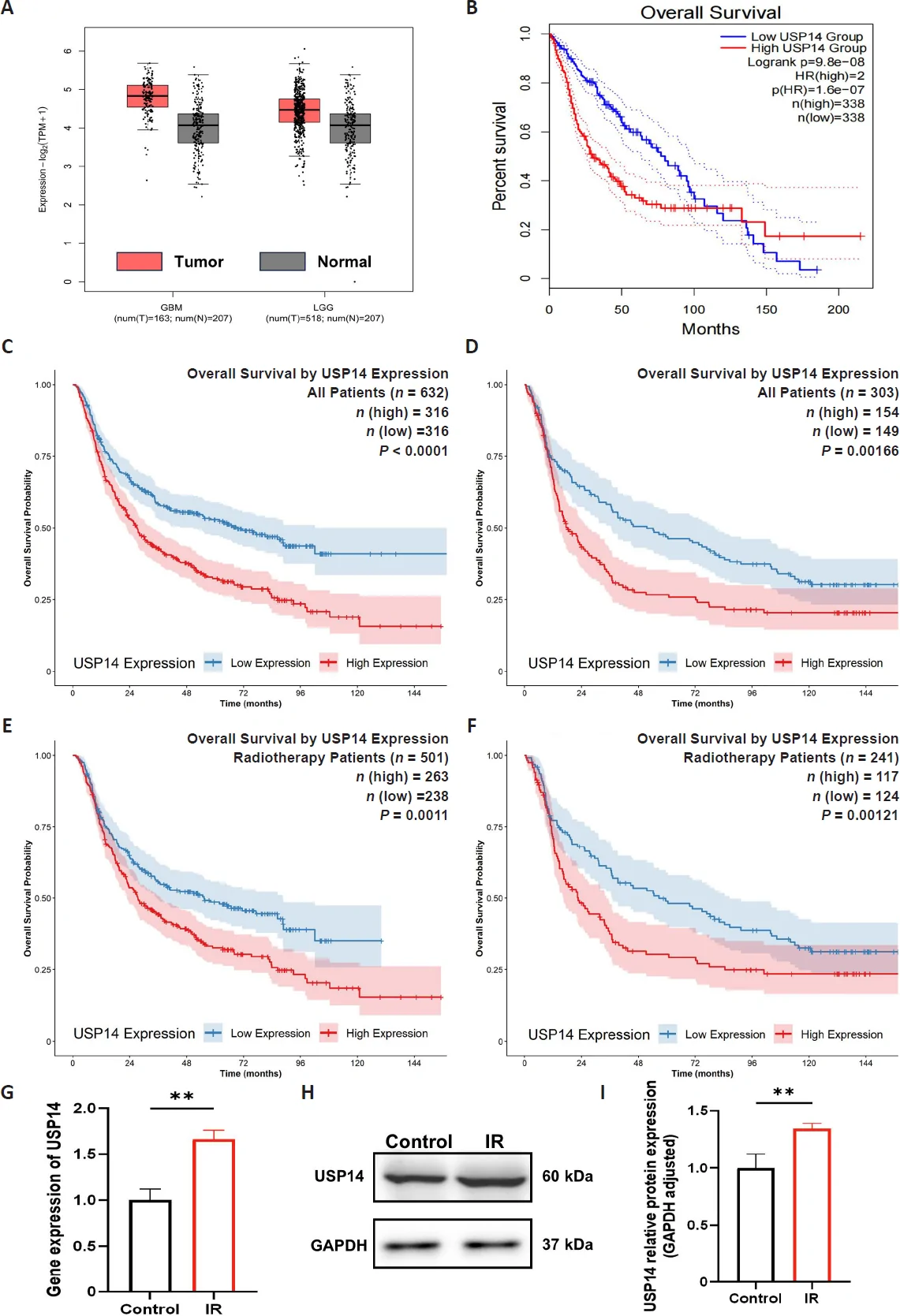
USP14 expression demonstrates prognostic value in glioma. (A) The USP14 expression profiles in normal brain, LGG, and GBM tissues in the Gene Expression Profiling Interactive Analysis database. (B) Kaplan-Meier plotter analysis assessing OS in glioma patients grouped by USP14 expression. (C, D) Survival analysis based on USP14 expression status in the Chinese Glioma Genome Atlas database. (E, F) Prognostic analysis of USP14 within the IR subgroup in the Chinese Glioma Genome Atlas database. (G) Expression of USP14 gene in the glioma patients with different treatments. (H) Western blot analysis of USP14 expression in C6 cells. (I) Quantitative analysis of USP14 expression. Data are expressed as mean ± SD (*n* = 3). **P < 0.01 (unpaired Student’s *t*-test). GAPDH: Glyceraldehyde-3-phosphate dehydrogenase; GBM: glioblastoma; IR: ionizing radiotherapy; LGG: lower-grade glioma; OS: overall survival; USP14: ubiquitin-specific protease 14.

### Inhibition of USP14 sensitizes glioma cells to radiotherapy

Given that high USP14 expression is associated with poor prognosis in glioma, we investigated its functional role in tumor progression using *in vitro* models. The b-AP15 was used both as a single agent and as an adjuvant to radiotherapy (**Figure 3A** and **B**), and its effects on cell viability, apoptosis, and clonogenic survival were evaluated. Treatment with b-AP15 at 10 or 20 μM for 24 hours significantly reduced glioma cell viability, and this effect was further enhanced upon combination with radiotherapy (**Figure 3C**). Live/dead staining further demonstrated that the combination treatment resulted in the greatest number of dead cells compared with either treatment alone (**Figure 3D**). Flow cytometric analysis of apoptosis revealed that while radiotherapy and b-AP15 each induced apoptosis individually, their combination produced a synergistic pro-apoptotic effect (**Figure 3E** and **F**). Specifically, the combination group exhibited a significantly higher apoptosis rate than either monotherapy or the control group, with the radiotherapy-alone and b-AP15-alone groups showing moderate yet distinct increases relative to untreated controls. Collectively, these data indicate that USP14 inhibition effectively sensitizes glioma cells to radiotherapy. Moreover, clonogenic assays showed that the combined treatment most potently suppressed the long-term proliferative capacity of glioma cells, further supporting the conclusion that targeting USP14 significantly enhances radiosensitivity (**Figure 3G** and **H**). Notably, the combination group yielded the fewest and smallest colonies, whereas the radiotherapy-alone and b-AP15-alone groups exhibited intermediate clonogenic suppression, and the control group showed the highest colony formation capacity.

**Figure 3:**
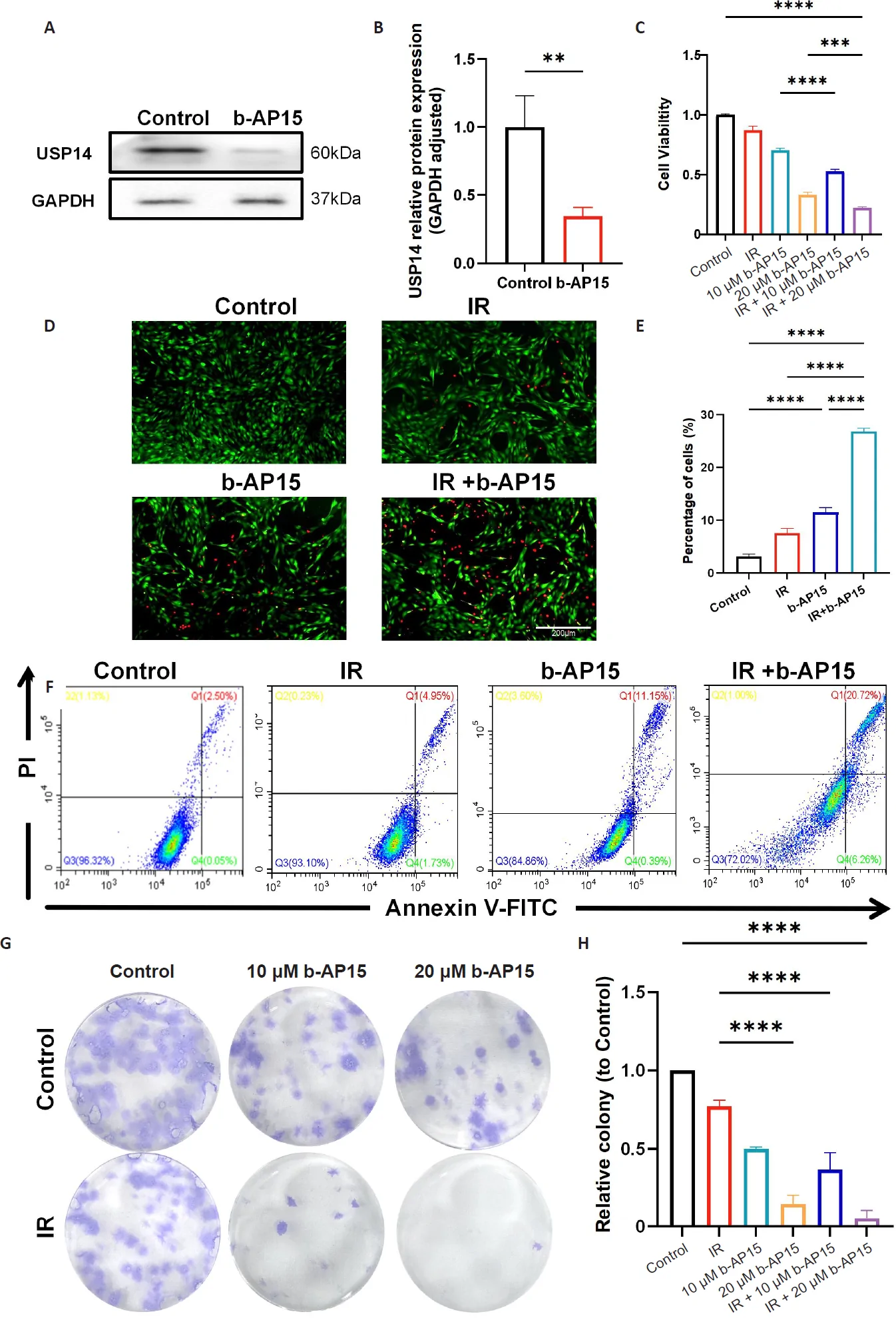
Inhibition of USP14 enhances the radiosensitivity of glioma cells (A) Western blot analysis of USP14 expression in C6 cells treated with 10 μM b-AP15. (B) Quantitative analysis of USP14 expression. (C) Cell viability (CCK-8 assay) of glioma cells under different treatments. (D) Live/dead staining of glioma cells across different treatment groups. Scale bar: 2.5 cm. (E) Bar graph of apoptotic cell quantification by flow cytometry. (F) Apoptosis analysis by flow cytometry in glioma cells from different treatment groups. (G) Clonogenic formation staining of glioma cells. (H) Quantitative analysis of Clonogenic formation staining. Data are expressed as means ± SD (*n* = 3 biological replicates). **P < 0.01, ***P < 0.001, ****P < 0.0001 (unpaired Student’s *t*-test (B), one-way analysis of variance followed by Tukey’s multiple comparisons test (C, E, H)). CCK-8: Cell counting kit-8; GAPDH: glyceraldehyde-3-phosphate dehydrogenase; IR: ionizing radiotherapy; USP14: ubiquitin-specific protease 14.

### Amplification and prolongation of radiotherapy-induced endoplasmic reticulum stress by USP14 inhibition

Having established that USP14 inhibition significantly sensitizes glioma cells to radiotherapy, we next investigated the underlying molecular mechanism. Since USP14 is a pivotal protein that controls UPS,^19^ we opined that inhibiting the protein would cause disruption in proteotoxic stress, and subsequently interfere with the ERS pathway, and as a result, induce the observed radiosensitization. To check this hypothesis, we subjected C6 glioma cells to b-AP15. The results of the western blot analysis revealed that 10 μM b-AP15 treatment resulted in a significant increase in the total ubiquitinated proteins, which indicated that ubiquitin homeostasis is disrupted and aberrant proteins accumulate as a result, which is a known ERS trigger. We next studied the expression of major ERS markers (**Figure 4A** and **B**). **Figure 4C–G** shows that treatment with b-AP15 alone increased the protein levels of phospho-PERK, phospho-eIF2a, ATF4 and CHOP, indicating the ERS. Notably, b-AP15 when used in combination with radiotherapy further and significantly upregulated the expression of these ERS-related proteins, which showed that the combination had a synergistic effect on the activation of the ERS pathway. In line with such results, quantitative polymerase chain reaction data revealed the considerable increase in the levels of PERK, EIF2a, ATF4, and CHOP mRNA upon the application of b-AP15, the highest magnitude of change of which was observed in the combination group, thereby validating the occurrence of ERS activation on the level of transcription (**Figure 4H–K**). These results align with the established role of USP14 as a key deubiquitinating enzyme in maintaining ubiquitin balance, whose dysfunction is closely linked to the initiation of ERS.

**Figure 4:**
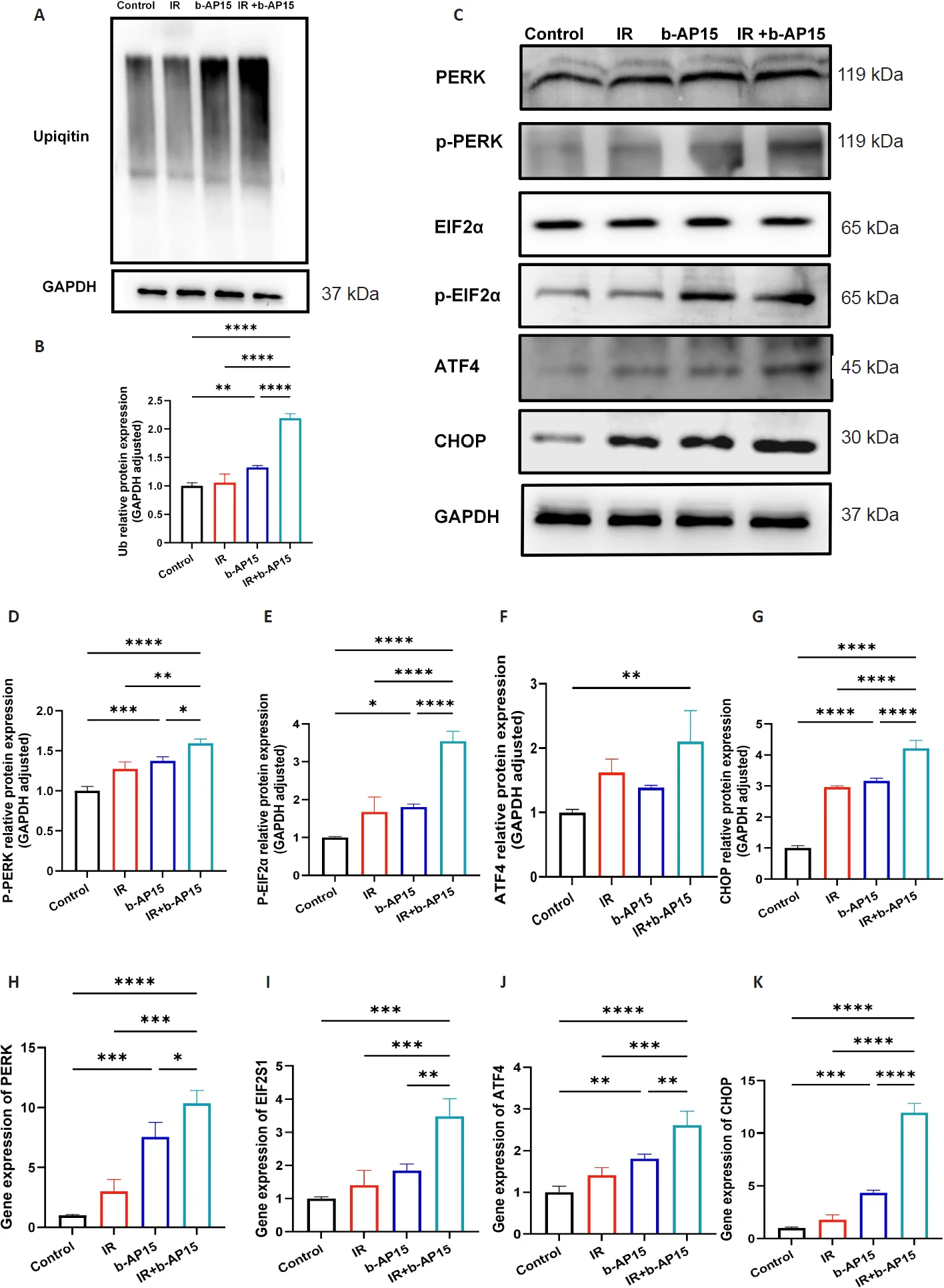
Amplification and prolongation of radiotherapy-induced endoplasmic reticulum stress by USP14 inhibition. (A) Western blot analysis of total ubiquitinated proteins in C6 cells treated with IR and/or 10 µM b-AP15. (B) Quantitative analysis of ubiquitinated protein levels shown in A. (C) Western blot analysis of phosphorylated PERK (p-PERK), phosphorylated eIF2α (p-eIF2α), ATF4, and CHOP. (D–G) Quantitative analysis of p-PERK, p-eIF2α, ATF4, and CHOP protein expression in C. (H–K) mRNA expression levels of PERK, EIF2S1, ATF4, and CHOP as determined by PCR. Data are presented as mean ± SD (*n* = 3 biological replicates). *P < 0.05, **P < 0.01, ***P < 0.001, ****P < 0.0001 (one-way analysis of variance followed by Tukey’s multiple comparisons test). ATF4: Activating transcription factor 4; CHOP: C/EBP homologous protein; EIF2S1: eukaryotic translation initiation factor 2 subunit 1; eIF2α: eukaryotic initiation factor 2 alpha; GAPDH: glyceraldehyde-3-phosphate dehydrogenase; IR: ionizing radiotherapy; PCR: polymerase chain reaction; PERK: protein kinase R-like endoplasmic reticulum kinase; p-eIF2α: phosphorylated eIF2α; p-PERK: phosphorylated PERK; USP14: ubiquitin-specific protease 14.

### Exacerbated endoplasmic reticulum stress results in reactive oxygen species accumulation and enhanced DNA damage

Given the marked activation of ERS, we next examined its downstream effects. Prolonged and excessive ERS can promote the overproduction of ROS through multiple pathways. As increased ROS levels and subsequent genomic DNA damage are critical mediators of radiotherapy-induced cell death, we investigated the impact of USP14 inhibition on ROS accumulation and DNA damage. Fluorescence staining revealed that both radiotherapy and b-AP15 treatment significantly increased ROS levels, with the combination treatment producing the greatest effect (**Figure 5A**). Immunofluorescence staining further showed an increase in γ-H2AX foci in cells treated with radiotherapy, b-AP15, or their combination, with the combination group exhibiting the highest number of foci per nucleus (**Figure 5B**). Quantitative analysis confirmed the elevation of ROS levels (**Figure 5C**) and the increase in γ-H2AX-positive cells (**Figure 5D**). These findings were further validated by flow cytometry in C6 cells (**Figure 5E** and **F**). As γ-H2AX is a well-established marker of DNA double-strand breaks,^20^,^21^ its upregulation in our experiments indicates that USP14 inhibition not only enhances ROS accumulation but also promotes DNA damage in response to radiotherapy. Together, these findings provide a mechanistic basis for the synergistic radiosensitization effect.

**Figure 5:**
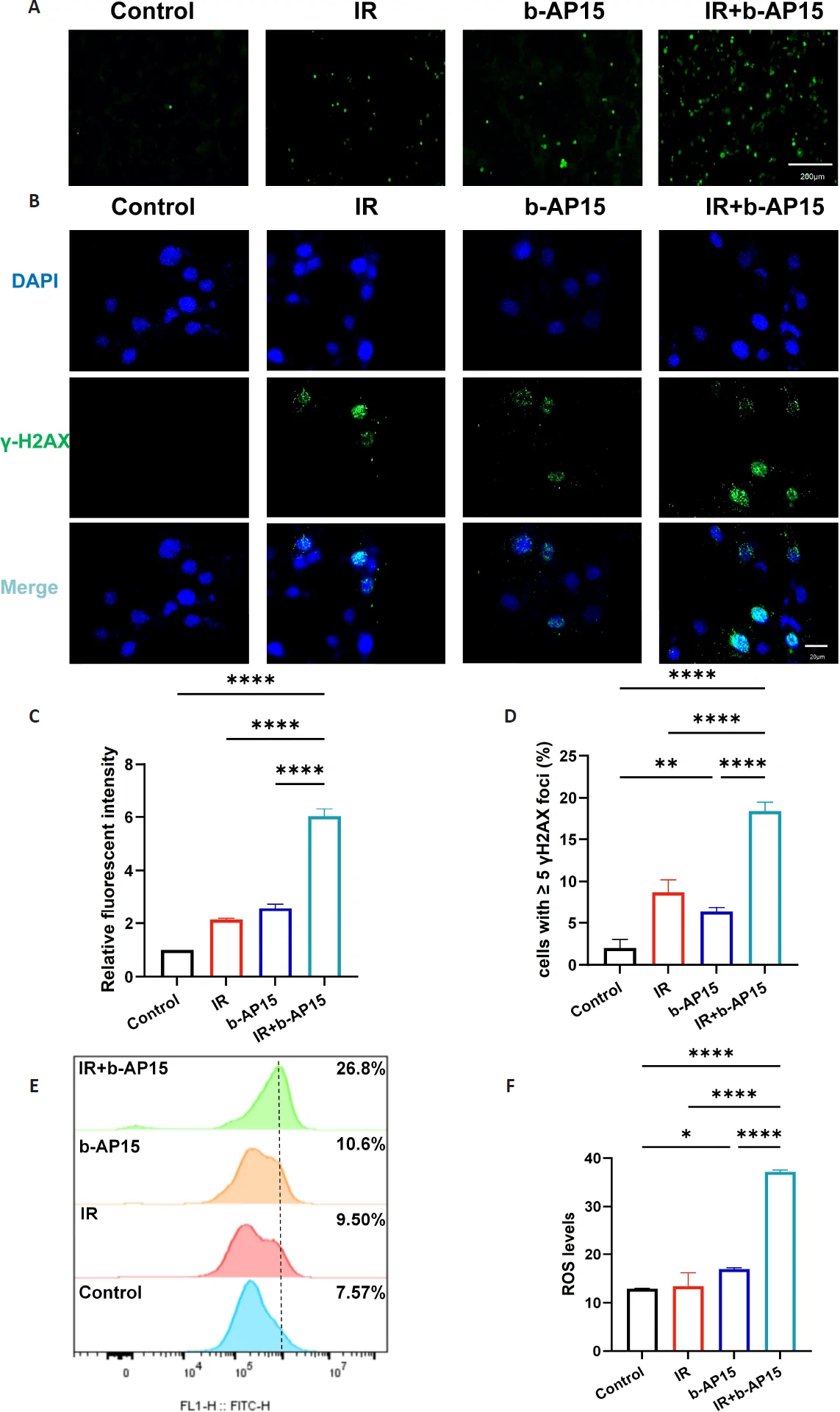
USP14 inhibition synergizes with radiotherapy to trigger ROS accumulation and subsequent DNA damage. (A) Representative fluorescence images of intracellular ROS levels in glioma cells under different treatments. Scale bar: 200 μm. (B) Representative immunofluorescence images of γ-H2AX foci (indicative of DNA double-strand breaks) in glioma cells. Scale bar: 20 μm. (C) Quantitative analysis of relative ROS fluorescence intensity from images in A. (D) Quantification of γ-H2AX-positive cells (defined as containing ≥ 5 distinct foci) from images in B. (E) Representative flow cytometry histograms of DCFH-DA fluorescence indicating intracellular ROS levels. (F) Quantification of the percentage of ROS-positive cells from flow cytometry analysis in E. Data are presented as mean ± SD (*n* = 3 biological replicates). *P < 0.05, **P < 0.01, ****P < 0.0001 (one-way analysis of variance followed by Tukey’s multiple comparisons test). DCFH-DA: 2′,7′-Dichlorodihydrofluorescein diacetate; IR: ionizing radiotherapy; ROS: reactive oxygen species; USP14: ubiquitin-specific protease 14.

### *In vivo* radiosensitization by USP14 targeting in glioma

Building on our *in vitro* findings that USP14 inhibition enhances radiosensitivity by activating ERS, increasing ROS accumulation, and exacerbating DNA damage, we next evaluated the therapeutic efficacy and underlying mechanisms of the USP14 inhibitor b-AP15 *in vivo* using an orthotopic glioma model in rats. The results demonstrated that combination therapy significantly inhibited tumor growth, as evidenced by tumor volume measurements and representative images (**Figure 6A** and **B**). To assess ERS activation *in vivo*, we examined the expression of the ERS marker GRP78 in tumor tissues and found that it was significantly elevated in the combination group (**Figure 6C** and **D**). ROS levels were assessed using the DCFH-DA fluorescent probe, which revealed the strongest green fluorescence in tumors from the combination group, indicating the greatest ROS accumulation (**Figure 6E**). Immunofluorescence staining for γ-H2AX further showed that the combined treatment induced more severe DNA damage (**Figure 6F**). Histopathological analysis additionally demonstrated that combination therapy significantly promoted tumor cell apoptosis and caused evident tissue damage with nuclear loss (**Figure 6G** and **H**).

**Figure 6:**
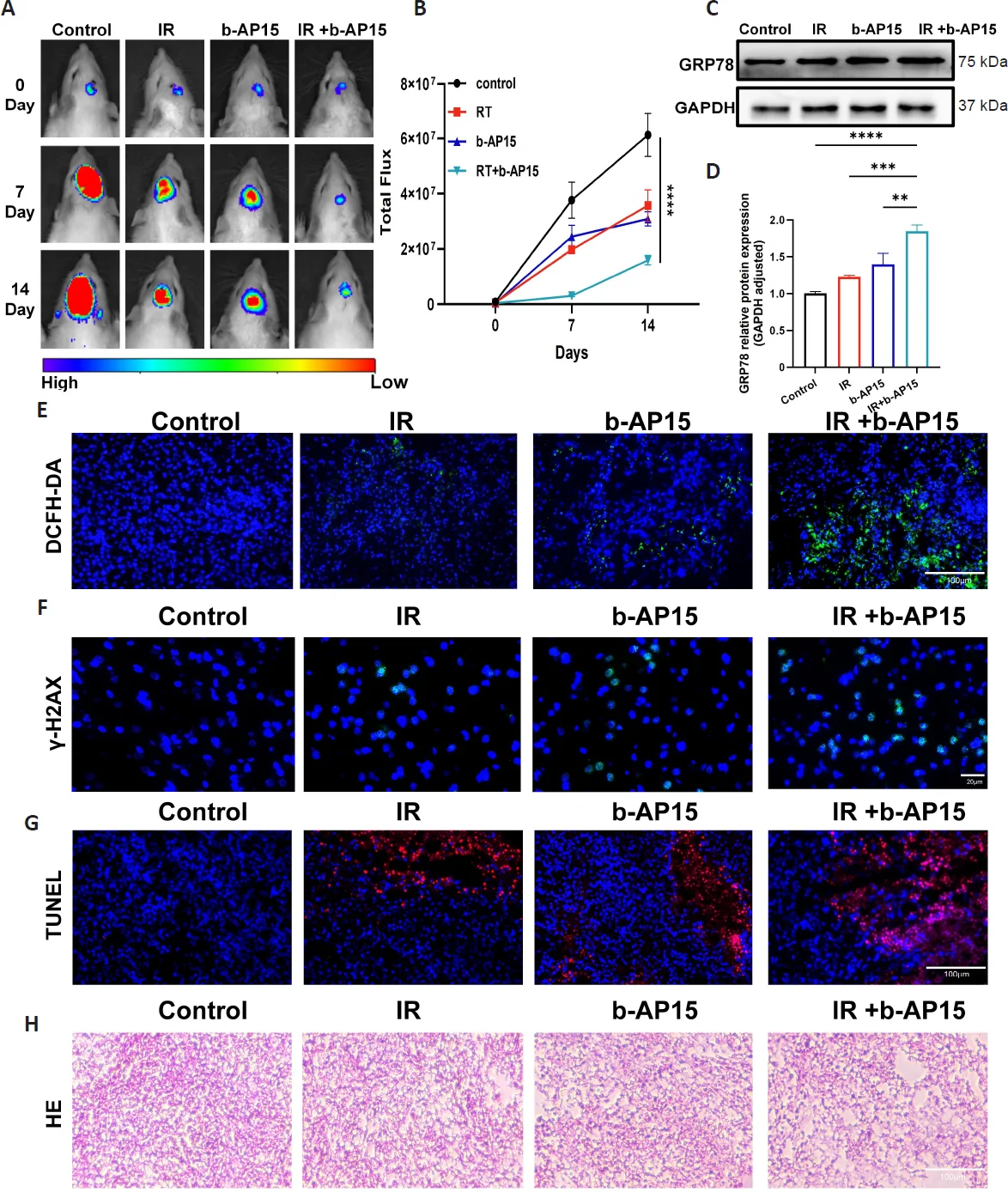
*In vivo* radiosensitization by USP14 targeting in glioma. (A) Representative *in vivo* bioluminescence images. (B) Quantification of total radiance flux from the bioluminescence imaging shown in A. (C) Western blot analysis of GRP78 in glioma tissues. (D) Quantitative analysis of GRP78 protein expression in C. Data are presented as mean ± SD (*n* = 3 mice per group). **P < 0.01, ***P < 0.001, ****P < 0.0001 (two-way analysis of variance followed by Tukey’s multiple comparisons test (B) or one-way analysis of variance followed by Tukey’s multiple comparisons test (D)). (E) Representative images of ROS in frozen glioma tissues. Scale bar: 100 µm. (F) Representative immunofluorescence images of γ-H2AX foci in glioma tissues. Scale bar: 20 µm (G) Representative images of TUNEL staining for apoptotic cells in glioma tissues. Scale bar: 100 µm. (H) Representative images of H&E staining of glioma tissues. Scale bar: 100 µm. GAPDH: Glyceraldehyde-3-phosphate dehydrogenase; GRP78: glucose-regulated protein 78; H&E: hematoxylin and eosin; IR: ionizing radiotherapy; ROS: reactive oxygen species; TUNEL: terminal deoxynucleotidyl transferase dUTP nick-end labeling staining; USP14: ubiquitin-specific protease 14.

Collectively, these *in vivo* results confirm that inhibiting USP14 effectively enhances the antitumor efficacy of radiotherapy in an animal model by aggravating ERS, promoting ROS accumulation, and intensifying DNA damage, providing compelling *in vivo* evidence for a radiosensitization strategy targeting USP14 in glioma.

## Discussion

The response of radiotherapy in glioma is inherently associated with the ability of the tumor to evade the oxidative impact created by the ionizing radiation.^22^,^23^,^24^,^25^ This adaptation goes beyond the activation of DNA repair machineries and includes the preservation of the proteostasis and redox equilibrium.^26^ In our study, it is observed that the deubiquitinase USP14 forms a critical node in this homeostasis. We are giving mechanistic evidence showing that the USP14 targeting in glioma cells brings about an extreme sensitization of the target cells to radiotherapy through cellular redox homeostasis disruption. This central process consists of USP14 inhibition causing proteostasis and ERS which is channeled into a significant accumulation of ROS. The radiation increases damages to DNA in accumulation of ROS, which eventually leads to apoptotic death of the tumor cell.

Over the past years, there has been an advance in the knowledge of the role of USP14 in glioma. A recent study has advanced our understanding of the multifaceted role of USP14 in glioma.^27^ USP14 has been shown to promote DNA repair and enhance radioresistance by stabilizing the demethylase ALKBH5. The present study further reveals an additional mechanism through which USP14 modulates radiosensitivity by regulating redox homeostasis. Specifically, we demonstrate that radiotherapy-induced upregulation of USP14 represents an adaptive response that buffers oxidative stress by mitigating the accumulation of ROS. Together, these findings position USP14 as a central hub integrating two critical determinants of radioresistance, DNA damage repair and oxidative stress adaptation. While DNA repair machinery addresses the consequences of radiation-induced damage, redox homeostasis mechanisms counteract oxidative stress through ROS buffering.^28^,^29^,^30^ Consequently, the induction of robust oxidative stress promotes a terminal pro-apoptotic state that, in turn, influences DNA damage to trigger cell death.^31^,^32^,^33^ Thus, USP14 supports glioma radioresistance not only by facilitating DNA repair but also by maintaining redox homeostasis, and its inhibition represents a promising strategy to redirect cellular fate toward death.

As a deubiquitinase proteasome-associated protein, the role of USP14 is more than just protein turnover.^34^ Our data, coordinating with the existing results, prove that high USP14 expression correlates with poor prognosis and radioresistance in glioma. Notably, radiation itself upregulates USP14,^35^ suggesting that USP14 induction forms part of a protective feedback mechanism against proteotoxic stress. Pharmacological inhibition of USP14 by b-AP15 resulted in marked accumulation of polyubiquitinated proteins, underscoring the essential role of USP14 in maintaining proteome quality. This disruption of proteostasis potently activated the PERK–eIF2α–ATF4 pathway of the ERS response. However, a crucial observation in this study is that this stress signaling does not lead to restorative adaptation. Instead, it serves as a trigger for redox dyshomeostasis.

We hypothesize that the synergistic radiosensitization observed upon USP14 loss-of-function converges on the dramatic accumulation of reactive oxygen species. ERS may enhance ROS generation through several interconnected mechanisms, such as the release of calcium from the ER lumen, which can subsequently disrupt electron transport in the mitochondria.^36^ Our experimental findings delineate a causal chain in which the combination of b-AP15 and radiotherapy leads to a marked accumulation of intracellular ROS, providing direct evidence for the synergistic effect observed. This oxidative surge was not an epiphenomenon but rather directly causal, as it led to the formation of γ-H2AX foci, marking a marked increase in DNA double-strand breaks.^37^ As a result, the apoptosis increased considerably and almost the entire survival of the clonogen was inhibited. Above all, the optimal antitumor effect of the combination therapy in the orthotactic model was associated with the strongest intratumoral ROS signal, most severe DNA damage, and most extensive apoptosis. These reproducible findings identify the ROS accumulation as an indispensable event bridging upstream disturbances in protein homeostasis with the downstream phenotype of radiosensitization.

This observation complements our knowledge of glioma radioresistance. Tumor cell survival following radiation exposure depends not only on efficient DNA repair but also on a robust redox buffering capacity that eliminates reactive oxygen species and clears damaged macromolecules.^38^ The USP14 is involved in this buffering capacity by protecting the proteasome activity. The inhibition of USP14 is an effective way to decrease the amount linked to the system, as well as the clearance rate of the latter, and make the oxidative flood occurring because of radiotherapy overwhelm a critical point to unleash irreparable damage. This model describes the strong synergy of co-targeting USP14 and radiotherapy.

Overall, this study incriminates the deubiquitinating enzyme USP14 as a critical factor of radioresistance in glioma. USP14 inhibition initiates a cascade comprising impaired ubiquitin homeostasis, followed by amplified radiotherapy-induced ERS and amplified ROS to aggravated DNA damage, which leads to radiosensitization of glioma cells at an extraordinary level. These studies depict the basic importance of USP14 in glioma radioresistance and provide viable opportunities to improve the treatment of glioma. Unlike traditional radiosensitizers that typically target a single pathway, inhibition of USP14 offers distinct advantages. First, it simultaneously disrupts multiple mechanisms of radioresistance, including the regulation of proteostasis, ERS, and ROS metabolism, thereby potentially limiting the adaptive capacity of cancer cells. Second, the USP14 inhibitor b-AP15 possesses single-agent activity, a favorable toxicity profile, and the ability to cross the blood-brain barrier, supporting its potential for clinical translation. Finally, the ER stress-ROS-DNA damage axis ultimately converges on a terminal cell death pathway. Collectively, these features position USP14 inhibition as a mechanistically distinct and highly promising radiosensitization strategy.

Several limitations should be acknowledged in this study. The *in vitro* experiments were primarily conducted using established glioma cell lines (e.g., C6), which may not fully capture the heterogeneity and microenvironmental complexity of patient-derived tumors. Additionally, the *in vivo* experiments utilized an orthotopic allograft model in immunocompetent rats, which does not fully reflect the human immune context or the pharmacokinetic and pharmacodynamic properties encountered in clinical settings. Beyond USP14, future studies will systematically interrogate other deubiquitinating enzymes within the broader ubiquitination network, such as USP5 and USP7, that may cooperate with or compensate for USP14 in maintaining proteostasis under radiation-induced stress. Systematic genetic or pharmacological targeting of these nodes, combined with radiotherapy, will help define their integrated roles in the glioma radiation response and reveal synthetic lethal interactions. Incorporating patient-derived xenografts, additional USP14-targeting strategies, and comprehensive toxicity evaluations will further strengthen the translational relevance of these findings.

## Data Availability

*The datasets generated during and/or analysed during the current study are available from the corresponding author on reasonable request.*
